# Behavioral sensitivity of Japanese children with and without ADHD to changing reinforcer availability: an experimental study using signal detection methodology

**DOI:** 10.1186/s12993-017-0131-6

**Published:** 2017-09-25

**Authors:** Emi Furukawa, Shizuka Shimabukuro, Brent Alsop, Gail Tripp

**Affiliations:** 1 Human Developmental Neurobiology Unit, Okinawa Institute of Science and Technology Graduate University, 1919-1 Tancha, Onna, Okinawa 904-0495 Japan; 2 Department of Psychology, University of Otago, Dunedin, New Zealand

**Keywords:** ADHD, Positive reinforcement, Signal detection, Japan

## Abstract

**Background:**

Most research on motivational processes in attention deficit hyperactivity disorder (ADHD) has been undertaken in Western Europe and North America. The extent to which these findings apply to other cultural groups is unclear. The current study evaluated the behavioral sensitivity of Japanese children with and without ADHD to changing reward availability. Forty-one school-aged children, 19 diagnosed with DSM-IV ADHD, completed a signal-detection task in which correct discriminations between two stimuli were associated with different reinforcement frequencies. The response alternative associated with the higher rate of reinforcement switched twice during the task without warning.

**Findings:**

Both groups of children developed an initial bias toward the more frequently reinforced response alternative. When the reward contingencies switched the response allocation (bias) of the control group children followed suit. The response bias scores of the children with ADHD did not, suggesting impaired tracking of reward availability over time.

**Conclusions:**

Japanese children with ADHD adjust their behavioral responses to changing reinforcer availability less than their typically developing peers. This is not explained by poor attention to task or a lack of sensitivity to reward. The current results are consistent with altered sensitivity to changing reward contingencies identified in non-Japanese samples of children with ADHD. Irrespective of their country of origin, children with ADHD will likely benefit from behavioral expectations and reinforcement contingencies being made explicit together with high rates of reinforcement for appropriate behaviors.

## Background

Attention deficit hyperactivity disorder (ADHD) is a common neurodevelopmental disorder characterized by elevated levels of inattention, hyperactivity and/or impulsivity that impair daily functioning. Upwards of 5% of elementary school-age children are diagnosed with ADHD [1], 50–65% of whom continue to meet diagnostic criteria for the disorder in adulthood [2, 3]. Attention deficit hyperactivity disorder has been identified across countries and cultures [4], with similar prevalence rates and symptom presentation, including in Japan [5, 6].

The disorder is known to be highly heritable (see [7] for review), however its precise etiology remains uncertain. Altered sensitivity to reward has been hypothesized to contribute to symptoms of ADHD (e.g., [8–13]). Evidence continues to accumulate in support of altered motivational processes in ADHD, although findings across studies are not entirely consistent, highlighting the complex nature of motivational processes [14]. At the behavioral level the most consistent finding is that children with ADHD are more likely to choose small immediate over larger delayed rewards compared with typically developing children (see [15] for a review). A number of studies report the performance enhancing effects of reward, on a range of cognitive tasks, are larger in children with ADHD than controls (see [16, 17] for reviews). Results from some studies also suggest the performance of children with ADHD is more similar to that of controls when reinforcement for correct responses is continuous or near continuous (e.g., [12, 18–22]).

Alsop et al. [16] recently demonstrated that children with ADHD are less sensitive to changing reward availability than typically developing children, when rates of reinforcement are low and changes are not signaled. Their findings are consistent with earlier reports that the behavior of children with ADHD does not match the reinforcement contingencies operating as well as that of controls [23, 24]. These findings may help explain the difficulty children with ADHD have in adapting their behavior to shifting situational demands in everyday life, where expectations for their behavior often change without warning (e.g., playing outside vs. sitting in a restaurant) and appropriate behavior is not continuously reinforced.

To date, the majority of studies assessing motivational processes in children with ADHD have been undertaken in Western countries. Only a small number of studies have evaluated reward sensitivity in children with ADHD from other cultural groups (e.g., South Africa [25], Japan [26], China [27]). Aase et al. [25] described reinforcers as being less efficient in establishing stimulus control in children with ADHD compared with controls in the Limpopo district of South Africa. Masunami et al. [26] reported Japanese children with ADHD paid more attention to reward than their typically developing peers while completing a variant of the Iowa Gambling Task. Most recently, Yu et al. [27] demonstrated Chinese children with ADHD show a stronger preference than controls for smaller immediate rewards over larger delayed rewards in temporal discounting and choice delay paradigms.

The results of these studies suggest the association between ADHD and altered reward processing is not a Western cultural phenomenon. However, further research is needed to confirm the presence of altered motivational processing in children with ADHD across cultures, preferably using the same paradigms so that findings can be directly compared. This becomes increasingly important as Western-styled behavior management programs are progressively adopted in non-Western countries [28].

In the current study we evaluate the sensitivity of Japanese children with and without ADHD to unequal frequency of reward and to changing reinforcement availability, using the signal detection task described by Alsop et al. [16]. Participating children were required to identify which of two stimuli were presented by making the appropriate response on a two-button response panel. The task began with one type of correct response being rewarded four times as often as the other. Such unequal arrangement of reward typically produces a response bias (preference) for the more frequently reinforced alternative [29, 30]; that is, the task examines the effects of reward on subsequent behavior, by exploiting instances of uncertainty regarding the correct discrimination between stimuli. In this study the ratio of available reinforcers for correct discriminations on the two response alternatives switched twice during the task.

Based on the findings of Alsop et al. [16], and the available evidence of altered reward sensitivity in children with ADHD in non-Western cultures [25–27], we predict the response allocation of Japanese children with ADHD will match reward contingencies less closely than that of their typically developing peers. Such an outcome would be of particular clinical relevance in Japan, where conformity is highly valued and the ability to adapt ones behavior to situational demands is especially important, but where behavioral expectations are seldom made explicit [31].

The overall sensitivity of the two groups of Japanese children to the asymmetric reward distribution is more difficult to predict. Praise is used sparingly in Japan [32–34] and how this experience will impact the children’s sensitivity to reward availability in the current task is unclear. Any differences in the reinforcement sensitivity of typically developing Japanese children, compared to their Western peers, would need to be considered in interpreting the data from the children with ADHD.

## Methods

### Participants

Data from 41 children, 19 meeting DSM-IV diagnostic criteria for ADHD (all boys) and 22 typically developing children (50.0% boys) living in Okinawa, Japan, are included in the study. Within the ADHD group, 10 children were diagnosed with inattentive type, one with hyperactive/impulsive type and eight with combined type ADHD. Four children were prescribed stimulant medication, which was discontinued for at least 24 h prior to study participation. Table 1 presents the sample characteristics.

**Table 1:** Participant characteristics

	Control			ADHD		
	(n = 22)			(n = 19)		
	Mean	sd	Range	Mean	sd	Range
Age (months)^a^	122.73	20.48	86–149	107.74	14.78	89–137
Estimated IQ	98.00	12.29	84–121	104.47	14.91	77–126
Boys, n (%)	11 (50%)			19 (100%)		
Stimulant medication (concerta), n	–			4		
Subtype, n: inattentive/hyperactivity_impulsivity/combined	–			10/1/8		
Comorbidity, n: oppositional defiant disorder				3		
Language/Tic/asperger disorder^b^				1/1/2		

^a^ The mean age was significantly higher for the control than ADHD group (*t*(39) = 2.65, *p* < .05)

^b^ Given that DSM-5 allows comorbid diagnosis of ASD with ADHD, children demonstrating symptoms of DSM-IV asperger disorder were included in the study if they satisfied other inclusion criteria. However, those demonstrating symptoms consistent with autistic disorder were excluded from the study due to accompanying cognitive impairments

Inclusion criteria for the study were estimated IQ scores above 70, participation in regular education classes^1^, normal or corrected vision, no past or current head injury, neurological disorder or psychosis. Comorbid conditions were allowed if these inclusion criteria were met. Children in the ADHD group were recruited through a university research clinic, where they completed multi-method, multi-informant research diagnostic assessments. Data from semi-structured diagnostic interviews (K-SADS-PL, disruptive behavior disorder section) [35, 36], parent and teacher completed rating scales for ADHD symptoms (SNAP) [37, 38], and observations of the child’s behavior were used to make a clinical diagnosis of ADHD. Parent and teacher completed broadband rating scales (CBCL/TRF) [39–42] and background questionnaires screened for other behavioral and emotional problems, neurological and medical conditions. Cognitive functioning was assessed with the WISC-III or WISC-IV [43–46].

Children were required to display six or more symptoms of inattention and/or hyperactivity/impulsivity in at least one setting, evidence of symptoms in a second setting, and functional impairment. Symptoms were not summed across informants. Assessments were carried out by a team including a US-licensed, Japanese-speaking clinical psychologist (EF), and clinicians with advanced counseling or other relevant degrees, all fluent in Japanese and experienced in working with children with ADHD. Control group children were recruited through invitation letters sent home to parents through public schools. These children completed an abbreviated IQ assessment (WISC-III Vocabulary/Block Design). Their parents and teachers completed the behavior rating scales, which were used to rule out the presence of ADHD or other significant behavioral or emotional disorders. Those demonstrating fewer than four symptoms of inattention or hyperactivity/impulsivity were included. Parent and teacher reports were reviewed for other inclusion criteria.

### Experimental task

**Fig. 1:**
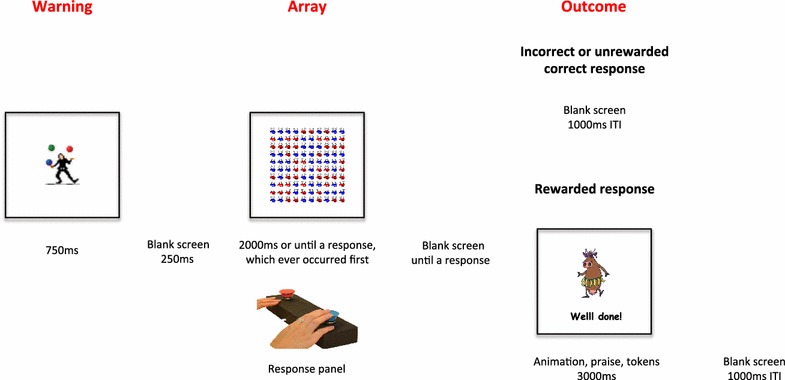
Experimental task timeline

The experimental task and procedure used is described in detail in Alsop et al. [16] and illustrated in Fig. 1. After instructions and a short demonstration of the task, children indicated whether there were “more red” or “more blue” characters in a checkerboard pictured on a computer screen (10 × 10 arrays containing either more red or more blue faces in a ratio of 54:46), using a two-button response panel. They were advised that correct responses only *sometimes* earn rewards. A multi-element reinforcement system was used to maximize reward effectiveness: the message “Well done!” and an animated cartoon appearing on screen, verbal praise from the examiner, and colored tokens placed in a clear plastic container next to the child. Following incorrect and non-rewarded correct responses, the screen was blank and the experimenter remained silent. All children received a prize at the end of the session irrespective of their performance on the task.

Successive blocks of eight trials contained an equal number of each array type, randomized within blocks. The computer determined quasi-randomly which correct responses were reinforced. At the start of the session, correct identifications of one stimulus (“more blue”) were rewarded four times more often than correct identifications of the other stimulus (“more red”). After the child had received 20 rewards with this distribution, the computer reversed the contingencies and correct identifications of “more red” stimuli were reinforced four times as often. After another 20 rewards, the original reward distribution was reinstated, with the game ending after the children received a further 20 rewards. Each successive block of ten reinforcements contained eight reinforcements for correct identification of one array type and two for correct identification of the other, randomized within each block.

### Data collection and analysis

Three measures of performance were calculated for each child: median response time, discriminability between stimuli (log*d*) i.e., accuracy, and response bias (log*b*) i.e., the systematic preference for the more frequently reinforced alternative [29].

Discriminability between the stimuli was calculated by the equation:

$$\log \;d = \frac{1}{2}\log \left( {\frac{{{\text{Blue}}_{Correct} }}{{{\text{Blue}}_{Incorrect} }} \cdot \frac{{{\text{Red}}_{Correct} }}{{{\text{Red}}_{Incorrect} }}} \right)$$

and response bias by:

$$\log \;b = \frac{1}{2}\log \left( {\frac{{{\text{Blue}}_{Correct} }}{{{\text{Blue}}_{Incorrect} }} \cdot \frac{{{\text{Red}}_{Incorrect} }}{{{\text{Red}}_{Correct} }}} \right)$$

where Blue_*Correct*_ denotes the number of correct responses following presentations of the “more blue” array, Red_*Incorrect*_ denotes the number of incorrect responses following presentation of the “more red” array, and so forth. Response bias scores were calculated relative to the response alternative that was reinforced more frequently during the initial phase. Mean response discriminability and response bias and median response time scores were calculated for all trials completed to receive reinforcements 1–10 and 11–20 (reward distribution 4:1), reinforcements 21–30 and 31–40 (reward distribution 1:4), and reinforcements 41–50 and 51–60 (reward distribution 4:1).

Mean scores for response bias, discriminability and median response time for the six blocks (2 blocks each for the initial, reversal and reinstatement phases) were analyzed with SPSS GLM. Mixed ANOVA was conducted to examine the main effects of Block (i.e., changes in bias, discriminability or response time over the 6 blocks) and Group (i.e., the difference in bias, discriminability or response time between the ADHD and control group), and the interaction effects. The bias scores were expected to change with the two shifts in the contingency schedule, therefore polynomial contrasts examined quadratic and linear trends in the bias scores over the six blocks, separately for the ADHD and control groups. Post-hoc pairwise comparisons were performed when significant univariate effects were observed. There were differences in the mean age and IQ, and gender, between the ADHD and control groups. Although the difference in IQ scores did not reach statistical significance, given the small sample size, IQ was entered as a covariate along with age and gender, when the two groups were compared on the performance measures.

## Results

### Response bias

**Fig. 2:**
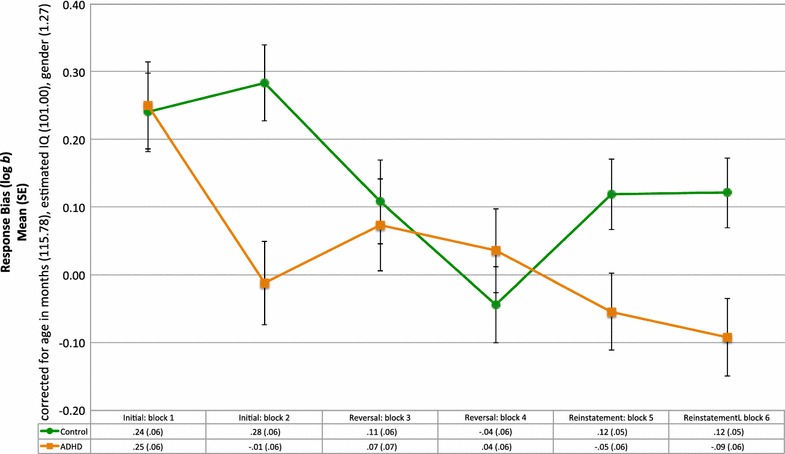
Mean response bias scores and standard errors for the ADHD and control groups during the initial, reversal and reinstatement phases

Mixed ANOVA indicated a significant univariate Block × Group interaction effect (*F*(5, 180) = 2.64, *p* < .05) for bias, controlling for age, IQ and gender (Fig. 2). For both groups, their bias scores were significantly different from zero during the first block (*p* < .001). The bias scores of the two groups were significantly different from one another during blocks two and six (*p* < .05, Bonferroni correction). Two polynomial contrasts were performed to assess quadratic and linear trends in the bias scores over the six blocks, separately for the control and ADHD groups. For the control group, there were significant quadratic (*F*(1, 21) = 9.50, *p* < .01) and linear (*F*(1, 21) = 12.02, *p* < .01) effects. Pairwise comparisons indicated a significant difference in the bias scores for the second and forth blocks (the second half of the initial and reversal phases; *p* < .01, Bonferroni correction). Mean bias scores decreased across both blocks of the reversal phase, increasing during the first block of the reinstatement phase. For the ADHD group, only the linear trend was significant (*F*(1, 18) = 31.68, *p* < .001). For this group the bias score for the first block was significantly larger than the bias scores for the two reinstatement blocks (*p* < .05).

### Discriminability

**Fig. 3:**
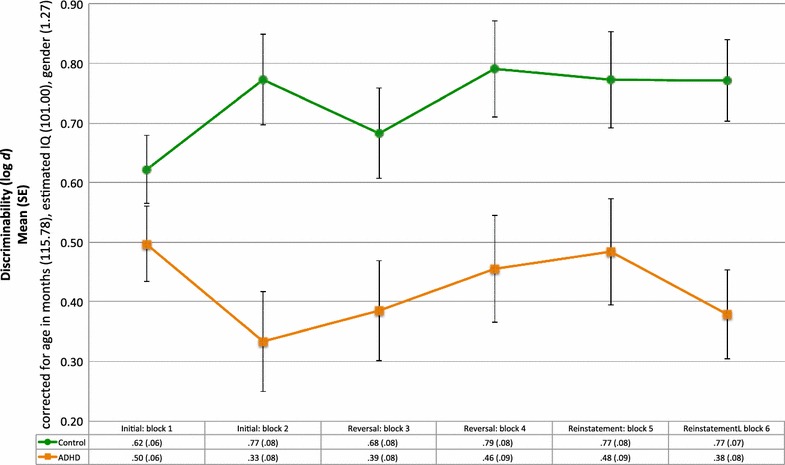
Mean discriminability scores and standard errors for the ADHD and control groups during the initial, reversal and reinstatement phases

Mixed ANOVA indicated a significant main effect of Group (*F*(1, 36) = 10.81, *p* < .01) after controlling for age, IQ and gender (Fig. 3). Discriminability scores were significantly higher for the control group for the second through sixth blocks of the task (*p* < .05, Bonferroni correction).

### Response time

**Fig. 4:**
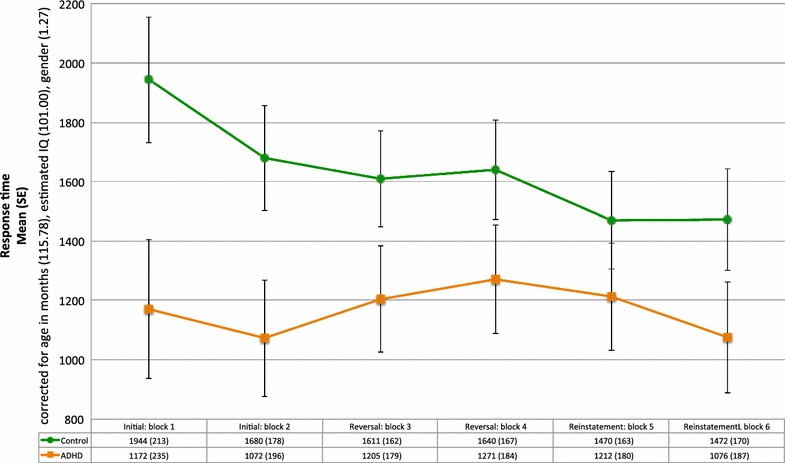
Median response time and standard errors for the ADHD and control groups during the initial, reversal and reinstatement phases

Mixed ANOVA indicated a trend toward a main effect of Group (*F*(1, 36) = 3.50, *p* < .10), after controlling for age, IQ and gender (Fig. 4). Pairwise comparisons show that children in the ADHD group responded more quickly than those in the control group during the first block of the initial phase (*p* < .05, Bonferroni correction).

## Discussion

The current study assessed the behavioral sensitivity of Japanese children with ADHD and their typically developing peers to unequal frequency of reward and changing reinforcement contingencies using signal-detection methodology. Both groups of children initially developed a preference (response bias) for the more frequently reinforced alternative, demonstrating their behavioral sensitivity to the asymmetric reward distribution.

As the task progressed the response allocation of the typically developing children reflected the availability of reward, i.e., as the reward contingencies changed the children’s response bias scores changed with them. The overall response pattern that emerged was very similar to that reported by Alsop et al. [16] for their New Zealand control subjects. Cross-culturally, typically developing children respond similarly to changing reinforcer availability, altering their response allocation to match the prevailing reinforcement contingencies. In both studies, the lower response bias scores seen in the reinstatement phase of the task likely reflect the children’s accumulated experience of reward over the initial and reversal phases of the task (i.e., their “reinforcement history” on the task). The similarity of the control group findings from New Zealand and Japan suggests the signal detection task is relevant to children from different cultural groups.

The response bias pattern of the Japanese children with ADHD suggests their ability to track changing reward availability is impaired. After developing a significant response bias toward the more frequently reinforced alternative during the first half of the initial phase, the children’s bias score dropped to near zero. This was followed by a slight increase in bias during the first half of the reversal phase, then a slow decrease across the remainder of the task. These data suggest a loss of behavioral control by the asymmetric reward distribution. Although the exact response pattern differed, Alsop et al. [16] also reported impaired tracking of reinforcement contingencies in New Zealand and American children with ADHD. In the current study, it is not clear why the children stopped responding to the asymmetric reward distribution so early in the task. However, the failure of the children to match their response allocation to the reinforcement contingencies operating is consistent with the difficulty children with ADHD have adapting their behavior when setting demands change (e.g., “often runs about or climbs in situations where it is inappropriate” [47]).

Poorer behavioral tracking of the reinforcement contingencies by the ADHD group does not reflect a lack of sensitivity to reinforcement. As noted, both groups of children developed a clear bias toward the more frequently reinforced response alternative early in the task. Nor does it reflect difficulty completing the signal detection task. Although discriminability scores were significantly lower for the children with ADHD, possibly reflecting less attention to the stimuli, their mean accuracy exceeded 70% across all phases of the task. The children with ADHD initially responded more quickly on the task than controls, the latter’s response times decreasing to a similar level with time on task. While the response speed of the ADHD group may have impacted their discriminability, it does not show a consistent relationship with bias scores, arguing against impulsive responding leading to poorer tracking of reward availability.

While the mechanisms underlying the poor alignment between reward availability and response allocation in children with ADHD remains to be determined, the current study provides further evidence that children with ADHD have difficulty adapting their behavior to situational demands when levels of reinforcement are relatively low and contingency changes are not signaled. We previously suggested such a disparity might arise as children with ADHD have difficulty adjusting their internal representations of reinforcement contingencies under low rates of partial reinforcement [8, 48]. Additional studies are needed to confirm the robustness of the observed effect together with theoretical and empirical research to address its underlying neurobiology. Theory-driven computational models could be developed to address questions regarding how prior experience with reward interacts with the prevailing contingencies to influence subsequent actions in those with and without ADHD. Alongside such theoretical work, neuroimaging and animal studies might examine frontostriatal activity patterns in response to changing contingencies during a conditional-discrimination task such as the signal-detection paradigm.

The overall similarity of the results to those reported by Alsop et al. [16], lends weight to the current findings. The study does, however, have limitations, including small sample sizes. The final ADHD sample is smaller than expected given the number of families who initially volunteered to participate. Compared with many countries, ADHD has a shorter history of recognition in Japan [5, 49]. This coupled with the stigma attached to mental health concerns may have increased the severity and complexity of the problems seen in children whose families volunteered for the study. Families of children experiencing pronounced difficulties may have been more willing to participate in research offering comprehensive multi-method, multi-informant assessments. The result being the exclusion of a number of children who did not meet the study criteria.

Groups in the current study were not well matched for age, IQ or gender, in part reflecting the challenges of data collection. As potential confounds, all three variables were included as covariates in the analyses, resulting in more conservative probability estimates, but no change in the findings. Importantly, within the control group there were no significant gender differences for age, IQ, response bias or discriminability.^2^ The assessment and diagnostic practices followed international guidelines and care was taken to rule out any psychiatric or neurodevelopmental disorders amongst the control group. We are therefore confident of group membership in our sample. In addition, the rate and pattern of comorbidity in the ADHD sample argues against other disorders explaining the current findings.

The signal detection task used in the current study has good ecological validity. Just as in children’s every day experiences, not all instances of correct behavior (i.e., successful discriminations) were rewarded and changes in reward availability were unsignaled [16]. These conditions may be especially relevant to the study of ADHD in Japan, where the use of praise is less normative [32–34]. As a more homogeneous population, societal roles are strongly tied to an individual’s identity and behavior [50], with children and adults expected to know the rules without them always being explicitly stated. Behavioral sensitivity to situational demands would be important in maintaining group harmony.

The present findings suggest similarities between Japanese and Western children with ADHD that go beyond symptom presentation. The results are consistent with reduced sensitivity to unsignaled contingency changes identified in non-Japanese samples of children with ADHD. Parents and teachers of Japanese children with ADHD should be advised of the importance of reducing ambiguity regarding behavioral expectations, irrespective of normative practices. Although Japanese culture has many daily rituals marking the transition from one setting to another (e.g., all students stand up and bow as the teacher enters the classroom), children with ADHD would benefit from explicit information regarding behavioral expectations in different settings and when these change. Furthermore, it should not be assumed that children with ADHD will carry this information forward to new environments, or that a failure to adapt behavior to situational demands is evidence of misbehavior. For children with ADHD, frequent, immediate and consistent reward for appropriate behavior is recommended across cultures.

## Conclusion

The current results provide additional evidence that children with ADHD do not track reward contingencies as consistently as typically developing children, when rates of reinforcement are low and the contingencies change without warning. The similarity of the findings, across studies and cultures, using the same paradigm and diagnostic procedures, offers further evidence for altered reward sensitivity playing a role in the pathophysiology of ADHD. Altered motivational processes may be a common, defining characteristic of the disorder expressed similarly across cultures. These findings also suggest recommendations to alert children with ADHD to situational expectations, and the consequences of their actions, are relevant across cultures. Further experimental research in non-Western countries, across the range of neurodevelopmental and psychiatric disorders, is important to confirm the cross-cultural continuity of core deficits and underlying neurobiology. Such efforts will help refine behavioral and pharmacological treatments and ensure their appropriateness for different cultural groups.

## Footnotes

1 Special education classrooms or day services in Japan tend to serve children with significant functional impairments.

2 The mean response time was faster for females throughout the task (*F* (1, 20) = 4.69, *p* < .05).
